# Mapping the Heartbeat of America with ChatGPT-4: Unpacking the Interplay of Social Vulnerability, Digital Literacy, and Cardiovascular Mortality in County Residency Choices

**DOI:** 10.3390/jpm13121625

**Published:** 2023-11-21

**Authors:** Mohammed M. Ali, Subi Gandhi, Samian Sulaiman, Syed H. Jafri, Abbas S. Ali

**Affiliations:** 1 Multidisciplinary Studies Programs, Eberly College of Arts and Sciences, West Virginia University, Morgantown, WV 26506, USA; mma0052@mix.wvu.edu; 2 Department of Medical Lab Sciences, Public Health and Nutrition Science, Tarleton State University, 1333 West Washington, Stephenville, TX 76402, USA; gandhi@tarleton.edu; 3 Department of Cardiology, Heart and Vascular Institute, West Virginia University, 1 Medical Center Drive, Morgantown, WV 26501, USA; samian.sulaiman@wvumedicine.org; 4 Department of Accounting, Finance and Economics, Tarleton State University, 1333 West Washington, Stephenville, TX 76402, USA; jafri@tarleton.edu

**Keywords:** CVD, artificial intelligence, large language model, CVD mortality, social vulnerability index, digital literacy

## Abstract

Cardiovascular disease remains a leading cause of morbidity and mortality in the United States (US). Although high-quality data are accessible in the US for cardiovascular research, digital literacy (DL) has not been explored as a potential factor influencing cardiovascular mortality, although the Social Vulnerability Index (SVI) has been used previously as a variable in predictive modeling. Utilizing a large language model, ChatGPT4, we investigated the variability in CVD-specific mortality that could be explained by DL and SVI using regression modeling. We fitted two models to calculate the crude and adjusted CVD mortality rates. Mortality data using ICD-10 codes were retrieved from CDC WONDER, and the geographic level data was retrieved from the US Department of Agriculture. Both datasets were merged using the Federal Information Processing Standards code. The initial exploration involved data from 1999 through 2020 (*n* = 65,791; 99.98% complete for all US Counties) for crude cardiovascular mortality (CCM). Age-adjusted cardiovascular mortality (ACM) had data for 2020 (*n* = 3118 rows; 99% complete for all US Counties), with the inclusion of SVI and DL in the model (a composite of literacy and internet access). By leveraging on the advanced capabilities of ChatGPT4 and linear regression, we successfully highlighted the importance of incorporating the SVI and DL in predicting adjusted cardiovascular mortality. Our findings imply that just incorporating internet availability in the regression model may not be sufficient without incorporating significant variables, such as DL and SVI, to predict ACM. Further, our approach could enable future researchers to consider DL and SVI as key variables to study other health outcomes of public-health importance, which could inform future clinical practices and policies.

## 1. Introduction

With the advent of computers, which progressively became more powerful over time, computing evolved from an arcane world where programmers wrote in computer code on punch cards fed to mainframes to a world where the computer interface is accessible on a smartphone’s screen. This evolution took many iterations to reach the current stage, where information can now be accessed using a feed with artificial intelligence (AI) running behind the scenes. Thus, ‘Artificial’ (nonhuman) intelligence techniques where training data uses backpropagation and ‘parameters’ to ‘learn’ the data and predict outcomes (discriminative artificial intelligence) and generate new output based on prior training and user input (generative artificial intelligence) was born. AI is, therefore, considered a branch of computer science that leverages intelligent entities to make predictions based on patterns in data employing algorithms [1,2].

One of the applications of AI has been in creating language models—AI systems that produce texts similar to humans based on machine-learning algorithms. Many AI tools, such as ChatGPT, chatbots, and language translation [1,3], are used in various fields for variable purposes, including the healthcare industry. ChatGPT responds to text-based inquiries from the audience to provide helpful responses to specific questions and comments. Particularly in medicine, this tool has been used by many healthcare professionals to obtain fast and accurate responses to health-related queries, hence accelerating the clinical practice guidelines [4]. As such, AI has greatly influenced the medical and biomedical research landscape today [1,2].

**Figure 1:**
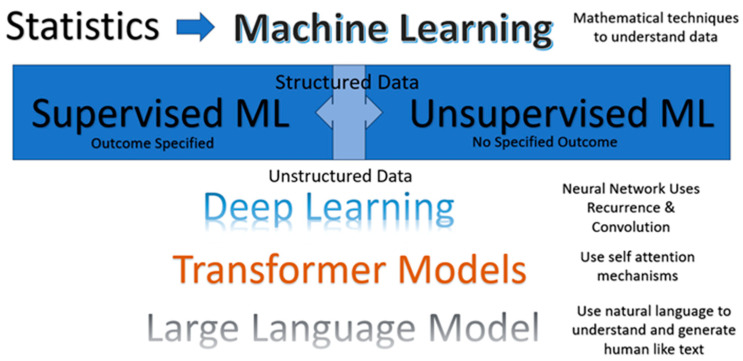
Types of Artificial Intelligence.

Statistics in particular uses mathematical assumptions, such as normal distributions and probability assumptions. Machine learning extends these ideas leveraging the power of computers to develop a ‘perceptron’ and run data through such conceptual structures multiple times. For example, a tree would repeatedly split data to reach a prediction; a random forest is a ‘forest’ of such trees that together capture the meaning of the data. In supervised machine learning, a known outcome against which predictions are calculated in part of the data (training data) and compared to actual outcomes in the output data. Consider nearest neighbors for unsupervised learning wherein several ‘centroids’ are initialized in data and ‘distance’ calculated from each centroid to each data point and the position of the centroid is adjusted until there is no further need for adjustment. Thus, data are partitioned into groups. Using the concept of eigenvectors, multiple variables are converted into principal components in principal-component reduction. Deep learning works on unstructured data using concepts developed in neural networks. These concepts are extended further in the sequential analysis of text data with the concepts of self-attention, and the entire corpus is considered at the same time in transformer models. These ideas are illustrated in Figure 1 below.

Medicine has always been at the forefront of seeking technological innovations in disease diagnosis and prevention. Naturally, AI was considered as one of the extrapolatory tools to improve patient outcomes [2,4]. Today, AI and language models are used as tools to analyze medical images, such as echocardiograms and angiograms, as well as to develop other interventions to improve the diagnosis and management of cardiac complications [1,5]. AI tools could also play a big role in identifying high-risk populations for hospital injury and guide interventions and preventive actions to improve patient safety [6]. Recent applications of AI in this domain have been in predicting hospitalizations and determining the mortality associated with COVID-19 [7,8]. The prospects and benefits of AI and machine learning use are well recognized and accepted in healthcare today concerning disease diagnosis, risk assessment associated with morbidity and mortality, surveillance of infectious diseases and outbreak prediction, and policy implications [8,9,10].

Although AI and machine-learning applications are gaining popularity in these arenas, the literature is scarce in recognizing their utility for predicting mortality using important determinants, such as digital literacy (DL) and the Social Vulnerability Index (SVI), significant predictors of any disease and all-cause mortality.

Many US adults use the internet to access information about health conditions today that are directly correlated with one’s overall well-being in multiple ways [11]. According to a recent nationwide survey, nearly 60% of US adults reported accessing health information online within the past year. The popularity of online access to information has increased over the years as no costs are involved in garnering such information [12]. Therefore, DL, which is defined as one’s ability to access, understand, and apply information retrieved digitally, is critical in reaching health equity, as it enables individuals from all backgrounds to access health information and medical terms through platforms such as computers and social media [13,14,15].

Similarly, the SVI is an important metric for forecasting survival according to the communities’ assets before, during, and after large-scale public-health disasters, such as hurricanes, tornadoes, and infectious outbreaks. The SVI index ranges from 0 to 1, with a higher value (close to 1) associated with lower income, higher poverty rates, and less access to educational resources, rendering communities vulnerable to disasters [16].

A stark illustration of the importance of social gradient is provided by an evident 48-year spread of life expectancy among countries ranging from 34 years in Sierra Leone to 81.9 years in Japan [17,18]. Similarly, in the United States, the death rate amongst the socially disadvantaged is triple compared to that of their higher socioeconomic strata peers [19,20]. The magnitude of the impact of social factors on physical functioning is similar to those of traditional risk factors (hypertension, diabetes, and smoking) [21], where increased deprivation of social privilege is associated with increased cardiovascular mortality [22]. An association between living in a socially advantaged area as opposed to a disadvantaged area has been previously described [20], showing a decrease in purchasing power to be associated with an increase in coronary artery disease and its risk factors. Additionally, socioeconomic status was found to favorably modulate the increased acute cardiovascular mortality conferred by exposure to particulate matter in the Phoenix area [23]. Different measures of social status have been used in the past to highlight the effect of disparity. For instance, Marmot [17], in their article, illustrates a gradient of mortality from the poorest 20% to the richest. Moissl et al. [20] used purchasing power as a measure, while Wilson et al. [23] used education and income. 

Machine-learning techniques have been found to identify novel predictors of cardiovascular outcomes [24] in comparison with conventional techniques, thus contributing to advancements in knowledge. Validation of unsupervised machine learning using principal-component reduction at identifying clinically relevant cardiovascular risk factors illustrates the potential of these modern techniques in identifying latent relationships [25,26] and adds another dimension to be considered- to expose heretofore unknown relationships. In other words, traditional statistics and supervised machine learning involve the generation of hypotheses and mathematical techniques such as linear regression. In unsupervised machine learning, abstract mathematical techniques and computing power are used to understand the underlying patterns in data. A prior study on the use of large language models (LLMs) on the UK biobank [27] exploits the ability of such models. A LLM projects words as vectors into a multidimensional space and identifies relationships using distance measures. Han et al. [27] illustrate how this can be accomplished by converting tabular data to text and feeding it to the LLM. Each row of their data is presented to the LLM as a sentence. LLMs exploit relationships found in languages or strings of words rather than tabular data, thus the format of the data submitted to the model was modified appropriately by the authors of this paper. LLMs also produce inconsistent results based on the system message, the input of the settings in the prompt, and the nature of the examples provided. In our analysis, we use measures of the SVI and DL with standard supervised machine-learning techniques.

For our analysis, we used the LLM to identify variables and generate code rather than to do the actual analysis of tabular data due to the known limitations of the LLM outlined above. Additionally, the Python code generated by the advanced analysis module of ChatGPT4 was verified by running it on a local machine with local data to verify consistent output.

**Figure 2:**
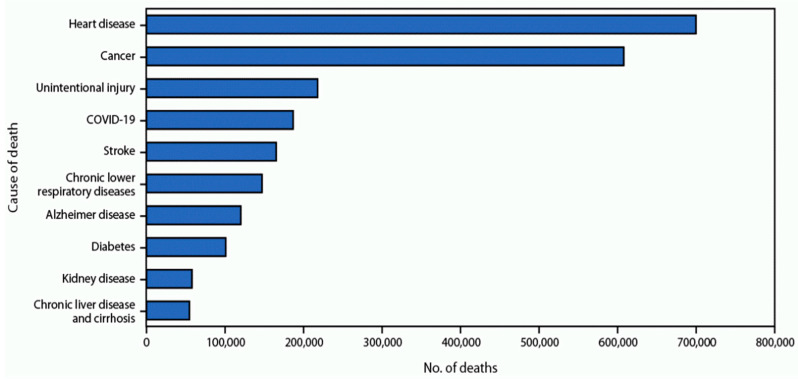
The top ten leading causes of death in the US (2022) [28].

Both SVI and DL can be considered in mathematical models leveraging machine-learning techniques to predict the disease-specific mortality rates for various geographic locations. However, machine learning has not been explored as a potential tool to predict cardiovascular mortality using these major predictors. This technique can be a useful tool to explore these leading causes of mortality using key predictors, such as SVI and DL, among others. The leading causes of death in the US are displayed in Figure 2.

The research question of interest for our study was whether SVI and DL predict variability in crude and age-adjusted cardiovascular mortality at the county level. Our study had two specific research objectives, as follows:

1. Evaluate if SVI and DL can significantly predict cardiovascular mortality for the general US population living in various states.
2. Evaluate the utility of AI tools, specifically ChatGPT, to predict cardiovascular mortality rates for different geographic locations.

## 2. Materials and Methods

### 2.1. Data Files and Merging

Freely available national databases were utilized to retrieve the variables of interest for our exploratory analysis. Crude mortality data was obtained from CDC WONDER [29], and the rural–urban county designation and years were obtained from the US Department of Agriculture [30]. The CDC WONDER website’s graphical user interface has several selection choices. The stepwise process of variable selection is listed in Table 1 below.

The Python census package, along with census API and the following Python code, allowed data on internet access and literacy to be downloaded easily. Digital literacy is a metric derived from this data. The abbreviated Python code used is presented below with key steps shown.

Data on social vulnerability is available at CDC/ATSDR and allows for the selection of year, state, and county. An excerpt from the Python code indicating the variable of interest chosen is presented below.

# Specify basic variables for age, gender, household type, and race

variables = [

   "B01002_001E", "B01001_002E", "B01001_026E", "B11001_002E",

    "B11001_007E", "B02001_002E", "B02001_003E", "B02001_005E", "B02001_008E"

]

These two files were merged using the Federal Information Processing Standards (FIPS) code (*n* = 3142). The FIPS codes are a standardized set of alphanumeric codes to ensure uniform identification of geographic entities such as states and counties in the United States [31]. Initial data exploration involved data from 1999 through 2020 from the CDC WONDER website for crude cardiovascular mortality using ICD-10 Codes: I00-I99 (diseases of the circulatory system) and county and rural–urban geographic categorization as choices. A linear regression line was fitted for change in crude cardiovascular mortality over time for the different rural–urban categories; the final data frame had 65,791 rows (99.98% complete data for 20 years for 3142 counties). For each year’s file, missing values were dropped before merging, which were checked at every step of the merging process; no missing values were generated. These data were used to develop model 1 [32]. The dataset used to calculate the final age-adjusted mortality rate using the same ICD-10 codes and geographic choices for 2020 had 3118 rows (99% of all US counties) with no duplicates, with 32 missing values for the age-adjusted rate and three missing values for the SVI and digital literacy (See Figure S1a,b in the Supplementary Materials). A ‘left merge’ was used in all merging instances using the mortality data as the file on the ‘left’; thus, all the available mortality data was preserved during the merge, and these data were used to develop model 2 [33].

SVI and DL as variables were merged using FIPS, resulting in a data frame to predict age-adjusted mortality. The final analysis was conducted considering a complete case analysis. After removing the rows with missing values (*n* = 32) in the ‘Age-Adjusted Rate’ column, we were left with 3118 observations for model development.

### 2.2. Selection of ChatGPT-4 and Python Integration

During the study, we used US governmental data, which, upon early exploration, was noted as having the primary drawback of being too broad—that is, it contained more variables than needed for our study. To address this issue, we selected an LLM (ChatGPT-4) to search the data for the defined parameters. By highlighting variables of interest through prompts, the LLM generated codes to build a comma-separated file.

The file was reviewed before being fed back to the LLM using the parameters to write codes based on the Python language. Code coherency was verified on a local computer by testing the code’s dataset against the source’s original dataset by looking at the analysis results.

#### 2.2.1. Important Consideration of Integration 

ChatGP-4 itself is in a ‘sandbox’ state and thus cannot directly access data on the internet. The user must provide access by uploading the data to a global cache. The data frame generated from running this code locally was then uploaded along with a prompt to ChatGPT4 after enabling advanced data analysis, a beta feature. All data generated by ChatGPT-4 was downloaded locally; any code generated by the LLM was run to verify and replicate the analysis.

##### Use of ChatGPT as a Beta Feature

ChatGPT4, being an LLM, is not particularly suited for the analysis of tabular data. The advanced data-analysis beta feature allows prompting of ChatGPT4, which, in turn, generates Python code using the advanced analytics and, in turn, uses the uploaded data file to generate the results requested. This Python code was then downloaded and modified to appropriately test on data on a local PC for the verification of results.

ChatGPT 4 was prompted to identify variables (see example ChatGPT4 prompt and response S1 in Supplemental Material). There are several methods by which one interacts with LLMs; these methods are named ‘prompting’. A prompt includes a system message and a user prompt. An early decision not to use Python to interact with ChatGPT 4 was made to keep the interaction with the LLM simple to follow. A paid ChatGPT 4 account was the foundation of the analysis. Such an account allows access to beta features, including advanced data analytics. Additionally, custom instructions were given to ChatGPT 4 to act as a data scientist, using census data, and to write output in the American Heart Association format. A prompt includes several technical components, including the maximum number of tokens, temperature, top-P, and frequency penalty. The techniques of the prompts include multiple-shot, few-shot, and single-shot techniques. This implies multiple examples of input and output, a few examples, or a single example of input and output. We used a single-shot technique for our study; that is to say, the prompt included an example of the output desired when appropriate.

Prompting continued to obtain information about crude and age-adjusted cardiovascular mortality data sources and variable names. Additionally, details on the socioeconomic county-level data variables available for download were sought. Once these files were obtained in a comma-separated format, further prompts to obtain Python code for data manipulation were obtained. Each output was then tested on the local PC and the output was assessed as to whether the desired data manipulation/statistical test was carried out correctly. Once a final clean data file was obtained, this was uploaded to ChatGPT4 after enabling advanced data-analysis tools with specific instructions on the step-by-step analysis to be carried out. The results were requested in word and comma-separated format.

#### 2.2.2. Python Package

Python package (version 3.10.9) ydataprofiling showed that all the numerical variables had a close-to-normal distribution; therefore, they did not need any transformations. Data were used as such without any particular feature engineering. Variables were chosen based on apriori knowledge of the subject rather than by using machine-learning tools. Thereafter, with the Python package sklearn.linear_model, we imported LinearRegression, Ridge, Lasso, ElasticNet, SGDRegressor, HuberRegressor, RANSACRegressor, and Lars. For model selection, sklearn.model_selection and traintest split were imported, and, from sklearn_metrics, mean_squared_error was picked. Data split using test_size 0.5 and random state 1 allowed us to run the models chosen above.

Using the particular model developed from the training-data mortality predicted with the test data allowed calculation of the mean squared error metric. This metric was used to compare the various models.

### 2.3. Data Integrity Assessment and Main Analytic Steps

Through the use of the Python language (version 3.10.9) ydata_profiling package to verify data integrity and quality, we were able to ensure sound reasoning. After validating the appropriate conversation of data, we were then able to explore the interaction effect between mortality and geographical areas (rural–urban) through a mixed-effects model with considerations (effects) for changes in mortality over the time studied (model 1), Figure S2a. A preliminary exploration of DL was made by analyzing SVI with reference to age-adjusted mortality at the county level using Python (model 2). Refer to Section 2.4.1 for a detailed description of the DL formula used in this study.

Changes in temporal trends in crude cardiovascular mortality for counties were explored using a mixed-effects statistical model to explore fixed and random effects of different geographic regions. This was necessary to address any potential bias when unifying multiple data groups in order to draw accurate conclusions. In this instance, variability in crude cardiovascular mortality in relationship to year and rural–urban classification (dependent variables) was modeled (model 1). Furthermore, several mathematical tools were vetted for age-adjusted mortality (linear regression, random forest, K nearest neighbors, and gradient boost) to identify the most robust model. We established that a third of the variation in age-adjusted cardiovascular mortality was accounted for by the two dependent variables (DL and SVI). Once this was accomplished, we used linear regressions using the most recent available data (2020) for age-adjusted cardiovascular mortality (2020). This analysis was implemented using linear regression (model 2).

### 2.4. Dependent Variables

Rural–urban status and year were the two dependent variables studied for the mixed-effects model (model 1). SVI and DL were the two dependent variables studied for the year 2020 to develop models studying variability in age-adjusted mortality (model 2).

#### 2.4.1. Calculation of DL Measure

DL as a dependent variable was used following the steps below:

1. No internet percentage [32] = Percent of households without internet in the county (B28002_013E: households without an internet subscription/B28002_001E: Total households) × 100;
2. No education percentage = Percent of households without education (B15003_002E: no schooling completed [33]/B15003_001E: total population 25 years and over) × 100;
3. Digital literacy DL = (100 − [‘No_Education_Percentage’]) × (100 − [‘No_Internet_Percentage’]). [Note—No educational attainment variable was derived from the American Survey [34] question “What is the highest degree or level of school this person has completed?” This is tabulated as B15003-002E in the data file. We adjusted the denominator to account for the overestimation of those without formal education.]

#### 2.4.2. Justification of DL Measure

**Figure 3:**
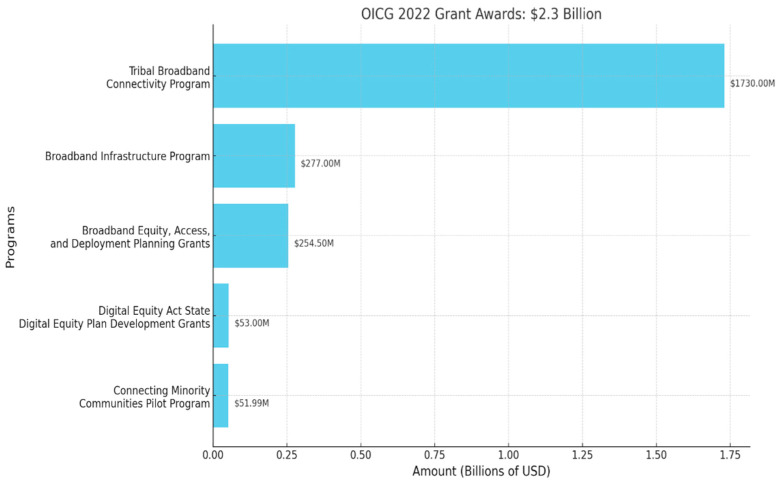
OICG 2022 Grant Awards [37].

In the European Union (EU), a measure of DL is the Digital Economy and Society Index (DESI), which tracks Europe’s digital performance [35]. The index includes five dimensions: (1) connectivity (fixed and mobile broadband, prices); (2) human capital (internet use, basic and advanced digital skills (see Figure 3)); (3) use of internet services (citizens’ use of content, communication, online transactions); (4) integration of digital technology (business digitalization, e-commerce); and (5) digital public services. The EU has standardized policies and procedures to monitor and track DESI. Rural dwellers with low levels of education and low socioeconomic status significantly lag in adopting the internet [36]. In 2010, the US Census started to gather specific information about the internet and data from surveys to assess internet access and education data as education is highly correlated with DL. Therefore, from the census, we used the inverse of “no internet access” measures and “no education” measures to estimate DL. The United States Office of Internet Connectivity and Growth spent less money on digital literacy in comparison to that spent on internet infrastructure [37] (Figure 3).

#### 2.4.3. How Literacy Contributes to DL

- Cognitive abilities: education equips individuals with the cognitive tools to comprehend, adapt to, and utilize new technologies [38];
- Access to resources: schools and colleges often provide computer labs, internet facilities, and formal information and communications technology (ICT) courses, giving students exposure and opportunities to become digitally literate [39]; Instructional experience: the structured curriculum in educational settings often includes components of DL, either embedded within subjects or as standalone courses [40]

### 2.5. Regression Model for Age-Adjusted Mortality for 2020 (Model 2)

**Figure 4:**
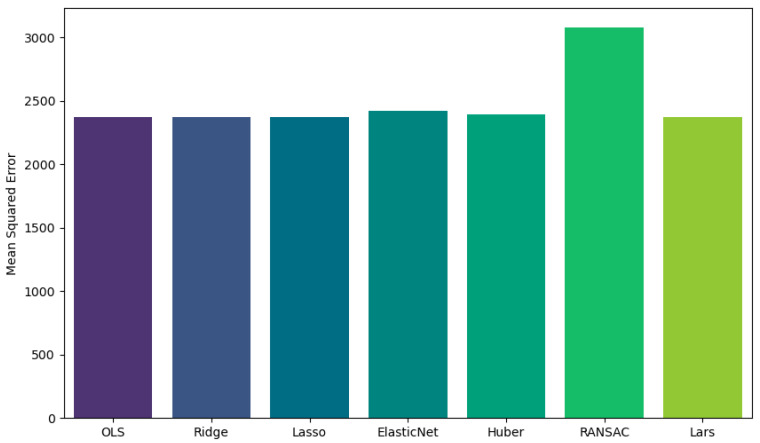
Mean square error calculation for various models illustrating similarity in the value of OLS to other complex machine-learning models.

A number of regression models were run (LinearRegression, Ridge, Lasso, ElasticNet, HuberRegressor, RANSACRegressor, and Larss), from which the ordinary least square (OLS) model was the simplest and best suited for interpretation purposes (mean squared error in Figure 4 below).

An exploratory data analysis revealed 99% complete data. Therefore, the missing values were simply dropped, and the outliers had plausible values and were used as such. We used two predefined features. Therefore, no feature selection processes were needed. Data split used a 0.5 setting. Hyperparameter tuning involved using the settings as follows: {‘OLS’: LinearRegression(), ‘Ridge’: Ridge(alpha = 1.0), ‘Lasso’: Lasso(alpha = 0.1), ‘ElasticNet’: ElasticNet(alpha = 0.1, l1_ratio = 0.5),’SGD’: SGDRegressor(max_iter = 1000, tol = 1 × 10^−3^), ‘Huber’: HuberRegressor(), ‘RANSAC’: RANSACRegressor(), ‘Lars’: Lars()}.

In general, machine-learning models are opaque/black boxes, while OLS models have interpretable coefficients. The equation for this simpler model without 2013 urbanization levels is below:

*Regression Equation*: *y* = 602.77 + 49.01 × *social vulnerability index* −4.51 × *Digital_literacy*

### 2.6. Assessing Best Regression Model Using Mean Squared Error as the ‘Loss Function’ (Lower Value Better) for Model 2

When considering geographic migration, individuals could potentially look at SVI and DL values to adjust for mortality data. In the OLS model, the prediction is made by substituting the values in the regression equation (shown below). The descriptors for model 2, predicting age-adjusted mortality with SVI and DL as dependent variables, are shown in Table 2.

*y* = *b*1 × *x*1 + *b*2 × *x*2 + *c*

Conversely, to generate a prediction using the LGBM regressor model, the input values must undergo preprocessing akin to what was done during the initial model training. After that, these preprocessed values must be fed into the computational model for execution. This procedure necessitates access to a computer with the requisite software to execute the mathematical model and the expertise to handle and format the data appropriately. These requirements pose technological challenges, unlike the simpler OLS model, where one can input data directly into a calculator using predefined equations.

### 2.7. Machine-Learning Steps for Age-Adjusted Mortality for the Year 2020

Executing machine-learning algorithms involves performing more than a thousand iterations to fine-tune the parameters for models like ‘lgbm’, ‘rf’, and ‘xgboost’ (for example, lgbmregressor), resulting in the creation of a thousand trees. Each iteration aims to rectify the mistakes made by the collective ensemble of models. With each iteration, the model recognizes features in terms of their importance and uses various techniques to adjust the loss function. Each iteration adds a new tree to the ensemble and is fitted to the residuals (errors) of the existing tree. The lgbmregressor model was chosen for its superior performance in regression analysis, evidenced by its lowest mean square error value of 2297. Within the lgbmregressor model, the DL feature importance was measured at eight and at five for theSVI. The OLS model mean square error value of 2373 was pretty close to the best model. We thus opted for the more concise OLS model to limit resource consumption and in the interest of ease of understanding.

### 2.8. Large Language Model—Chat GPT4 as a Detective Agency

ChatGPT4 is a LLM [41] based on transformer architecture and consists of two main parts: an encoder and a decoder. The encoder converts the input text into dense vectors or embeddings and considers the position of each word (positional embedding). A portion of the encoder focuses on different parts of the input sentence (multihead attention); these outputs are passed to feed-forward neural networks. Layer normalization and residual connections form the rest of the encoder structure. The decoder has an output embedding with a structure similar to that of the encoder. The first step is to break down the prompt into a sequence of tokens, which are the smallest units the model can understand. These tokens are then embedded into vectors representing them in a high-dimensional space. Each token then considers other tokens (self-attention) in the input when giving a response. Each attention output is fed into a feed-forward neural network that applies learned transformations, and this passes through multiple such layers. Only a certain number of tokens can be considered (context window); higher scores are assigned to tokens more relevant to the query, and an output is generated. The investigative layers of language models summarizing these steps are displayed in Table 3.

#### The Detective’s Context

The output is based on the clues provided, the detectives’ expertise (trained parameters), and their collaborative reasoning (attention mechanism and neural networks). If this case is part of a broader investigation (such as naming multiple variables in a dataset), the detectives keep a ‘case history’ to ensure their suggestions are consistent and contextually appropriate, similar to how GPT-4 maintains context in a conversation (Table 3). Therefore, using natural language prompts and directing ChatGPT4 to find the variables of interest to create specific tables becomes a simple process.

**Figure 5:**
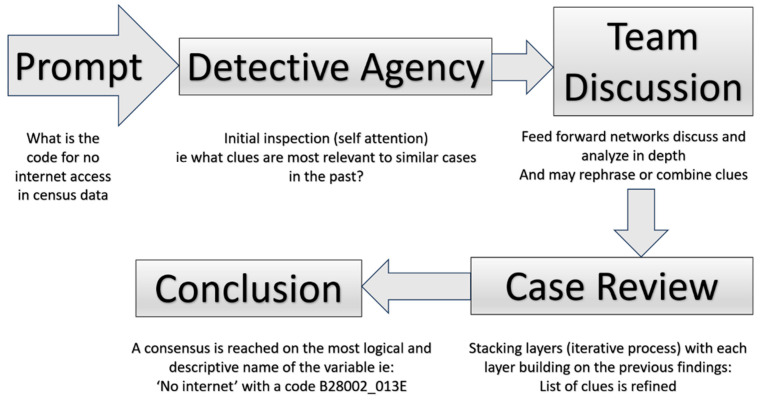
ChatGPT4 acting as a detective agency to access information about “No Internet Access”.

An example of how ChatGPT4 acts as a detective agency to find information about internet use from the census data is demonstrated below in Figure 5.

## 3. Results

### 3.1. Rural–Urban Level Analysis of Crude Cardiovascular Mortality Data (1999 to 2020)

#### 3.1.1. Exploratory Data Analysis Leading to Model 1

The regression and RUCC1 models for rural–urban categories suggest clearly identifiable trends, indicating that they are not properly capturing variability in the 1999 to 2020 crude cardiovascular death-rate data (see Figure 3 above). Furthermore, the polynomial regression model and the RUCC have different baselines and slopes, suggesting a change in the model from regression to a mixed-effects model (model 1). Please see Figure S2a,b, and Table S1 in the Supplementary Materials.

#### 3.1.2. Mixed-Effects Model of Crude Cardiovascular Mortality Data (1999 to 2020, Model 1)

**Figure 6:**
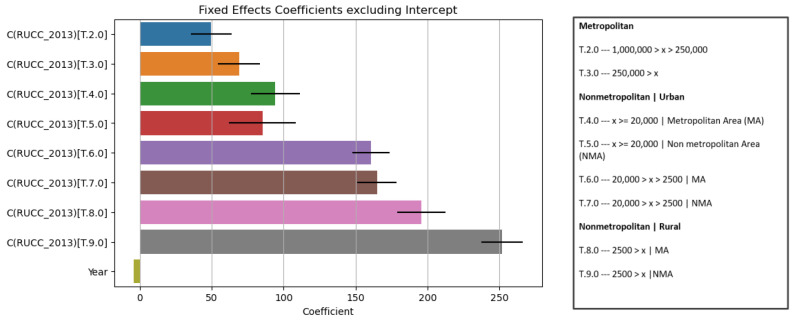
Mixed-effects model with metro areas (population ≥ 1 million) as the base case.

Data available from 1999 to 2020 were represented geographically to support the crude death rates. We observe that the rates decreased from nonmetropolitan to metropolitan and rural to urban areas (Figure 6). Each category follows the US Census Bureau’s urban–rural designations [34] (also represented in Figure 6’s key to the right of the graph).

#### 3.1.3. Comprehensive Regression Model for Age-Adjusted Mortality for 2020 (Model 2)

**Figure 7:**
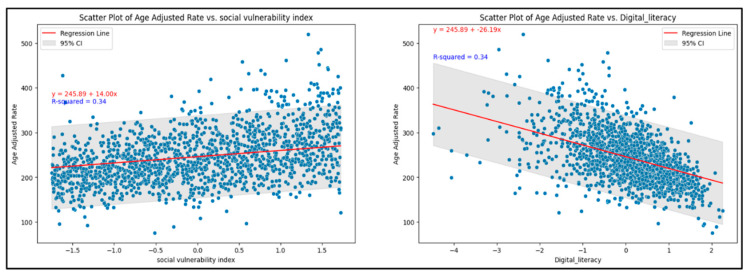
Relationship of DL and SVI with age-adjusted mortality. [Note: Equation and R squared annotated in each figure for the partial model].

**Figure 8:**
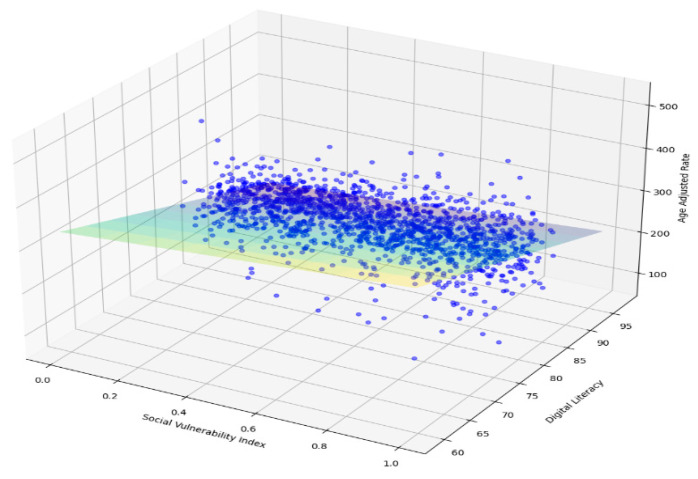
Performance of the OLS Regression Model, the blue dots represent the data points and the light blue-green plane the mathematical model fitted to the data.

**Figure 9:**
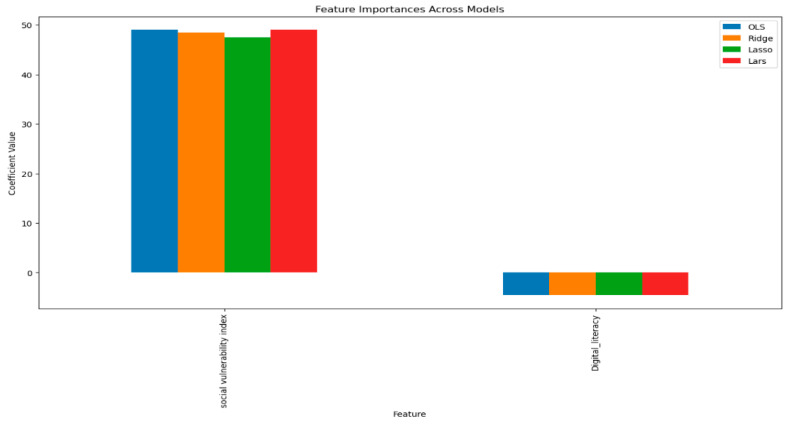
Features of figure importance for various models extracted from each model.

The exploratory data analysis revealed an inverse relationship between age-adjusted mortality and DL and a positive relationship between SVI and age-adjusted mortality (Figure 7). Multiple regression models (Linear Regression, Ridge, Lasso, ElasticNet, HuberRegressor, RANSACRegressor, and Lars) were used, and, from these models, the OLS had an acceptable performance (Figure 8). The features of importance of the models are illustrated in Figure 9. The R-squared value of the OLS simple model was 0.34, implying that the model captured 34% of the variability in age-adjusted mortality. The SVI contributed 6% (R squared 0.06) while DL was 18% (R squared 0.18). These R-square values do not add up to 0.34 for the full model due to several possibilities, including interactions, nonadditive effects, and the presence of multiple predictors; due to mathematical constraints, the R squared of the reduced model is always greater than that of the complete model. Thus, the partial R squares do not sum up. The 3D graph in Figure 8 shows the combined relationship between DL, SVI, and the age-adjusted rate for the year 2020. This graph represents the entire linear regression model.

All the models rated the DL and SVI as features of importance and the coefficient values are similar. Therefore, it would not particularly matter as to which model would be selected, as they all seem to have derived a similar ‘understanding’ of the data.

#### 3.1.4. Regression Model for Age-Adjusted Mortality for 2020 (Model 2a)

When a separate model was run that incorporated 2013 urbanization levels, the *p*-value coefficients were not significant (equation below), implying that this variability in the rural–urban divide is successfully captured by DL and SVI (Table 4).

[*y* = 540.43 + *b*1 × *x*1 + *b*2 × *x*2 + *b*3 × *x*3 + *b*4 × *x*4 + *b*5 × *x*5 + *b*6 × *x*6 + *b*7 × *x*7 + *b*8 × *x*8 + *b*9 × *x*9 + *b*10 × *x*10]

#### 3.1.5. Age-Adjusted Mortality Prediction per County Using Model 2 and Machine Learning

The age-adjusted mortality rates using the two regression methods (OLS and LGBM regressor) are demonstrated in Table 5, with a random selection of counties. As demonstrated, the age-adjusted rates can be easily predicted using the SVI and DL measures to make informed choices about moving to another county. Certain interesting counties defying expectations are listed in the supplemental methods; these counties would be expected to have high mortality and yet did not (Table S2).

## 4. Discussion

In this study, we aimed to demonstrate the practical application of an LLM, specifically ChatGPT4, and its benefit to health-outcomes research. Because CVD is the leading cause of death in the United States [28,42], and underserved areas usually experience higher mortality rates [28,42], utilizing machine-learning tools such as LLM can help in understanding the geographic imbalances linked to CVD mortality. The rationale was to use existing public data from the available quality public resources to feed queries on the variables desired into ChatGPT4 to see how closely it would help identify the variable names needed. Then, the rationale was to use ChatGPT4 to generate Python code to download data and upload the processed data back to ChatGPT4 to see how this aligns with the current literature in explaining the variability in mortality due to robust determinants, such as DL and SVI. Through ChatGPT4, we successfully demonstrated that LLMs can adequately help demonstrate the relationship between the key determinants, such as DL and SVI, in predicting CVD-related mortality for various geographic locations.

### 4.1. Innovation in This Study

We used ChatGPT4 to identify variables of interest from large national databases, such as CDC WONDER and the US Census, to study the relationship between the explanatory variables (DL and SVI) and the outcome of interest (CVD mortality), which has not been explored to date to our knowledge. For example, the American Community Survey has roughly 31,000 variables for one-year data and 16,000 for five-year data [43], with one-year data available for geographic areas with 65,000 people, while the five-year data was combined to produce data on geographic areas with fewer than 65,000 people. The adoption of ChatGPT allowed us to search through 16,000 variables in negligible time, which would have been a labor-intensive process if done with traditional methods. As an LLM, ChatGPT 4 can be relied on to capture the ‘essence’ of the variables and their meaning; thus, if we prompted it by asking for a measure of internet access, it could be relied upon to ‘understand’ our query and generate the correct response.

SVI considers multiple factors, such as poverty, lack of access to transportation, and crowded housing, that contribute to a community’s ability to prevent human suffering and financial loss in the event of disaster [16,36]. Historically, SVI has been a key measure to study the management and response to disasters [44]. Chakraborty [45] and his team demonstrated that socially vulnerable populations are disproportionately exposed to flood risks. The application of SVI is also popular in the area of infection control [46], where its application allows for the implementation of control measures (e.g., isolation, masking, and contact tracing) during pandemics such as COVID-19. Only a handful of studies have used SVI as a determinant to study CVD prevalence using national databases [47,48,49,50] using traditional statistical methods.

DL encompasses a range of skills, from basic understanding and usage of computers and internet usage to more complex tasks such as coding and digital content creation [14,38]. Users must possess DL to access digital medical services [15]. As such, DL is increasingly recognized as a key competency in the 21st century, especially in underserved areas, as a tool to narrow the equity gaps [13,39].

Through LLMs, we adopted a highly efficient technique to expedite the research process and provide the data to the scientific community at a much faster rate compared to the traditional methods, which could also help the practitioners in identifying determinants and making prognosis decisions for patient outcomes. To our knowledge, this is the first study to use an LLM to demonstrate geographic variation in CVD-related mortality using SVI and DL as predictor variables.

### 4.2. Strengths of Our Study

We sourced high-quality, publicly available data, as mentioned above. After our data processing and merging steps, we were able to retain more than 99% of all US counties in our final analysis. The data accessible from CDC WONDER [29], one of our data sources, is considered the gold standard for these reasons: (i) standard data-collection methods, (ii) rigorous peer-reviewed methods, (iii) ease of geographic search and access to downloadable data for FIPS, (iv) timeliness for data archiving, and (v) adhering to ethical guidelines for research protocols [51,52,53]. Similarly, our second data source from the US Census [30,54] demonstrates similar characteristics with regard to its (i) transparency and reproducibility, (ii) quality control and checks, (iii) high geographic area granularity with access to FIPS codes, and (iv) availability of data via an application programming interface [55,56,57].

LLMs are being widely considered for application in medicine, as they can respond to free-text queries without being specifically trained in the task in question However, the popularity of LLMs is mixed, with excitement and challenges [58]. In this study, the investigators devised a plan to use an LLM, specifically ChatGPT4, to bring attention to a major public-health issue—CVD-related mortality. Hence, we attempted to use ChatGPT4 to explore its utility for public-health research [41]. We successfully demonstrated that tools like ChatGPT4 can be quite efficient compared to traditional analytical methods, proving its utility in health-outcomes research. Further, with the use of machine-learning tools, we demonstrated that determinants, such as DL and SVI, can be used as predictors to study geographic-specific CVD-specific mortality rates.

Automated machine-learning prediction of cardiovascular outcomes correctly identified 368 more cases of first fatal or nonfatal myocardial infarction than the Framingham Score [59]. In the proposed study, we followed an approach similar to that adopted by Goldstein and colleagues [60]. These authors illustrate the benefits of machine-learning approaches in handling nonlinear data, reducing the number of predictors, and explaining various techniques.

The authors involved in this study were the subject-matter experts and statisticians with a vast knowledge of machine-learning techniques. When the LLM created errors due to various known LLM pitfalls, reading the code made it easily apparent and fixable for the team. One common issue was that the LLM failed to import the necessary Python packages and assign numerical values to variables before executing the commands. Despite these challenges, the LLM generated several functions and newer data-analysis methods using Python and often suggested better and more aesthetic graphics than those conceived by the authors. The authors benefited from the ‘collective wisdom’ of the various coding strategies the LLM was trained on and were able to provide high-quality visuals for the readers.

While we used LLMs to identify the variables of interest, we adjusted the model and the data throughout our analysis to minimize biases. For example, using the standard tabular data approach in these models, we took additional steps to identify variables identified by the LLM and generate codes which were then applied to the data analysis. Furthermore, for each year’s file, missing values (which biases the results in LLM models) were dropped before merging. In addition, given that the data was for 20 years for the first model, there were only 32 missing values for the crude death rates. For the data used from 2020 for the second model, barely three values for the SVI and DL rates were missing, which offers confidence in the results derived. At every iteration of the analysis, a detailed evaluation of the preliminary results was analyzed, adjusted, and then fed back into the empirical model to reach the results presented in the study. Finally, as machine-learning models are opaque/black boxes and the mean square error measure of model fit was similar to the ordinary least squares (OLS) regression, this model was used for testing the relationship between the dependent and independent variables. The principal results from the analysis, namely that death rates are higher in nonmetropolitan areas than metropolitan and higher in rural areas than urban areas, indicate the potential impact of low DL rates and a higher SVI on the increased CVD mortality rates in the underserved rural and nonmetropolitan areas (see Figure 6, Figure 7 and Figure 8). Our results validate similar results and recommendations obtained with previous studies included in the review of the literature section [13,15].

The summary of the strengths of our analysis includes (i) the robustness of our underlying data, (ii) the reproducible codes, (iii) the varied analytic methods used, and (iv) the simplicity of the final model chosen. Data quality is maintained systematically by either the CDC or the U. S. Census using excellent data-curation processes to ensure quality. The predictors (DL and SVI) were carefully chosen to predict adjusted CVD mortality. For example, SVI adequately encapsulates social vulnerability and is a comprehensive measure chosen by the CDC [16]. Additionally, our feature-selection processes did not use machine-learning techniques, and the completeness of our data approached 100%. Thus, we analyzed quality data from quality sources that did not need data-imputation techniques due to their completeness, thus producing reliable and reproducible results.

### 4.3. Limitation of Our Study

Admittedly, there are limitations to the study, given the scope of our study and the available resources. For example, even though we included age-adjusted death rates as the dependent variable for the second model and limited the regressors to the DL and SVI as regressors, we do recognize that the model has identification problems. Mortality due to CVD is also influenced by a shortage of specialists, such as cardiologists in the medically underserved areas [61,62], low insurance rates among socioeconomic and rural groups (although these are accounted for in the SVI), and transportation issues, among others [63]. Since our study is at a population level (not at an individual level), granular county-level data for traditional risk factors is difficult to come by and not addressed in our study. As our study was specifically focused on DL and SVI, we do not claim that the results represent an overall and comprehensive representation of the causes of mortality associated with CVD. As previously recognized [64,65], “while ChatGPT allows huge datasets to be analyzed in a short (relatively) period of time, there are limitations and constraints”, such as computational calculations constraints, the potential for inaccuracies, and inadequate inferential capability. Our research using state-of-the-art methods attempted to address several issues related to the use of the ChatGPT model even within the confines of the aforementioned limitations. Additionally, we adopted a ‘blended approach’, wherein a LLM was used specifically for two purposes, namely, to help narrow down the variables of interest and, secondly, to help generate and correct the Python code. Thereafter, a comparison of numerous machine-learning models helped us pick OLS as the method of choice. Thus, the strengths of the LLM, wherein it is able to identify ‘closeness’ in high dimensional space, complemented the interpretability obtained from the statistical techniques (i.e., OLS), giving a suitable result.

Census data is sparse for places with a population of less than 65,000, which may have omitted more vulnerable populations in predicting CVD-specific mortality. Urban counties were overrepresented when DL was considered as the variable of interest in the study. When data for the RUCC categories was explored, it became apparent that the definitions of RUCC were revised every decade, making comparisons across time less reliable. While there was no significant change in classification (the national percentage of rural and urban populations remained consistent with the 2010 definition), the underlying population shift by the new definition could have introduced bias in our current findings [66]. Even though there has been a demonstration of good prediction with AI models when studying mortality and hospitalization rates for certain health conditions such as COVID-19, there could be concerns about replicability and the bias inherent in the study [7], including ours.

#### 4.3.1. Potential Biases

There are potential biases that may have arisen in our study due to multicollinearity, aggregation bias, and model-related issues. Inherent biases from the data ChatGPT is trained on and the curse of dimensionality (as the dimensions increase, data becomes sparse) exist in our analysis.

Our exploratory data may be prone to multicollinearity. For example, people with limited access to the internet are associated with low educational attainment, tend to live in rural areas, and are more likely to face adverse health outcomes. These factors were not controlled in the study. Further, the internet-access data was retrieved at the household level without accounting for individual factors (e.g., someone living in the same household who may have access to the internet). A careful study by Lavery and Acharya concludes that the presence of multicollinearity increases Type-II error (false positive) but is unrelated to Type-I error [67]. Similarly, a power analysis is needed to infer causality [68] Furthermore, there is a potential bias in using aggregate bias in large datasets, as the aggregation of data fails to capture important differences in subgroups. A prior study [69] in analyzing the relationship between mortality rates of the broader Hispanic population due to CVD discovered that when aggregated data were used as they were in previous studies, the relationship between the Hispanic population and CVD-related deaths was insignificant. However, by disaggregating the data by subgroups within the Hispanic population, the rates were unique to each of the subgroups. Van Smeden et al. [65] spell out a detailed list of questions (12 in number) and biases inherited in using predictive modeling in the medical literature. Gleaning through this article, our study addressed several of the issues, although a few biases may still remain.

#### 4.3.2. Generalizability

Even though our study has a nationally representative sample, caution should be exercised during its interpretation. However, our results validate other studies which link mortality rates influenced by DL [70,71]. To overcome the access issues for patients, especially for rural residents and health education in the general population, additional resources—both in the development of communication infrastructure (broadband access) and technical literacy educational programs with the masses—being needed is a generalization or a broader application of this research.

### 4.4. Public Health and Policy Implications

Cardiovascular diseases such as heart disease and stroke are the leading causes of death in the United States, contributing to nearly one third of all fatalities. In addition to the loss of life, these diseases pose a significant burden on healthcare costs, reaching USD 216 billion in annual expenditures [72,73]. Even though medical technologies have improved patient health outcomes, social and behavioral determinants cannot be ignored in the equation to improve cardiovascular health outcomes at a population level. For example, over 90% of cases of myocardial infarctions can be prevented by addressing modifiable risk factors [74]. LLMs such as GhatGP4 are already revolutionizing the field of medicine with their advanced power to detect diseases and create fine images [75]. If optimized and utilized with awareness of their biases and error rates, such AI-based applications could accelerate the identification of social, economic, and behavioral factors for designing and implementing interventions that influence CVD-specific mortality.

The most recent Infrastructure Investment Bill and American Jobs Act (IIJA) has earmarked USD 65 Billion for broadband projects to close the digital divide, with USD 43 billion appropriated for the Broadband Equity Access and Deployment Program, or BEAD program, which will significantly contribute to the expansion of US broadband infrastructure [76] In addition, the states also initiated funds to reach out to vulnerable populations, including those who primarily reside in rural areas. This study has demonstrated that, while broadband access is crucial for reaching out to underserved populations, it is a necessary but not sufficient condition to improve access to digital resources and health and wellness information [77]. If individuals want to utilize health information from the web, they require digital health literacy to make informed decisions. Historically, underserved populations, such as minorities and those with low socioeconomic status, do not have optimal DL to access their electronic health records [78]. These are the vulnerable populations that also live in higher SVI areas [16]. In examining patterns among users and nonusers of electronic health records, Mukhopadhyay and his team [78] discovered that encouragement and push from the providers can “nudge more patients towards utilization of digital care or health services”. At a broader level, digital training and strengthening local “digital champions”, individuals or organizations such as public-health officials and the network of United States Department of Agriculture extension agents, can play these roles “who help, identify, design, and deliver skills training] for underserved populations [79]”.

## 5. Conclusions

In this study, we demonstrated that incorporating DL and SVI, among other variables, is essential in predictive modeling, as these factors can largely explain disease-specific mortality for individuals residing in various parts of the country. Additionally, we demonstrated that using an LLM, such as ChatGPT4, can greatly expedite similar analyses for specific health outcomes that pose a significant public-health burden. This approach can be instrumental in implementing interventions to improve health outcomes by informing policies.

## Figures and Tables

**Table 1:** Selection of key variables for analysis using CDC WONDER data.

Headings under Request Form Tab on wonder.cdc.gov (Accessed on 31 October 2023)	Data Selected
1. Organize table layout:	County 2013 urbanization categories Measures (for model one—Crude rate, for model two—Age-adjusted rate)
2. Location	Individual states Aggregate data for the United States All 2013 urbanization categories
3. Demographics	All demographic data (e.g., age groups, gender, Hispanic origin, single race 6)
4. Select year and months	Appropriate year(s) chosen
5. Weekday autopsy and place of death	All weekdays Autopsy—all values Place of death—all places
6. Select Cause of death	Select the ICD-10 choice. Diseases of the Circulatory System (I00, I99)

**Table 2:** Descriptors of regression model 2.

Description of Variables	Coefficient (β)	Intercept
*y* (Age-adjusted mortality)	NA	*c* = 602.77
*x*1 = SVI	49.01	
*x*2 = DLI	−4.51	

**Table 3:** Investigative Layers of ChatGPT4 acting as a Detective Agency.

	Process	Example	Description	Simili
ENCODER	User Prompt leads to tokenization	“What is the Code for no internet in the census?”	Tokenization involves breaking down sentences into smaller units known as tokens. The tokens are compared to model-trained parameters. Self-inspection identifies the crucial text.	This is similar to the first detective who reviews clues and consults the agency’s database to find the most relevant clues compared to past cases
	Feed Forward Network		Identified tokens are analyzed, with some being combined and others discarded.	This is analogous to a team of detectives refining the case based on prior cases.
	Stacking layers		An iterative process, each layer builds on the findings of the previous layer using attention mechanisms and backpropagation.	This is similar to clues being continuously refined in terms of their significance.
DECODER	Conclusion		The process concludes by suggesting a variable name as the output.	This is similar to a process in which detectives reach a consensus and generate an output.
	Contextualization		The model considers the ‘*n*’ most recent tokens to keep the process in context.	This is similar to a detective who keeps the case history, especially when the investigation is part of a larger context.

**Table 4:** The descriptors of the regression model.

Description of Variables	Coefficient (*β*)	*p*-Value
*y* (age-adjusted mortality)		
*x*1 = SVI	50.28	0
*x*2 = DVI	−3.83	0
*x*3 = RUCC 2 (metro areas with a population of 250,000 to 1,000,000)	0.11	0.975
*x*4 = RUCC 3 (metro areas with a population of less than 250,000)	0.59	0.86
*x*5 = RUCC 4 (nonmetro area adjacent to a metro area with a population of 20,000 or more)	7.67	0.053
*x*6 = RUCC 5 (nonmetro area not adjacent to a metro area with a population of 20,000 or more)	0.41	0.94
*x*7 = RUCC 6 (urban population of 2500 to 19,999 adjacent to a metro area)	11.44	0
*x*8 = RUCC 7 (urban population of 2500 to 19,999 not adjacent to a metro area)	3.92	0.23
*x*9 = RUCC 8 (completely rural or less than 2500 urban population adjacent to a metro area)	4.23	0.316
*x*10 = RUCC 9 (completely rural or less than 2500 urban population not adjacent to a metro area)	4.08	0.265

Note: The boxplots indicate coefficients for the various RUCC_2013 categories, a larger coefficient indicates greater importance. The legend on the right specifies RUCC_2013 categorizations and number of people in each category. Base population ≥ 1 million.

**Table 5:** Estimates of age-adjusted mortality using the OLS Model and LGBMregressor.

	Social Vulnerability Index	Digital Literacy	Predicted Age-Adjusted Mortality Rate per 100,000 (OLS Model)	Predicted Age-Adjusted Rate per 100,000 (LGBMregressor)
Greene County, Ohio	0.16	92.17	232	240.83
Midland County, Michigan	0.15	88.2	247.89	241.4
Polk County, Minnesota	0.4	88.02	260.62	242.6
Mariposa County, California	0.83	87.01	285.95	242.6
Grand Isle County, Vermont	0.01	94.85	213.44	237.4

## Data Availability

Data from CDC Wonder and the US Census Bureau were made available for free download.

## Supplementary Materials

The following supporting information can be downloaded at: https://www.mdpi.com/article/10.3390/jpm13121625/s1, Prompt and Response S1: Example of a ChatGPT 4 code generator prompt, an illustration; Figure S1. (a) Description of Outlier Counties and Counties with Missing Values. (b) Counties with Missing Values for the Age-adjusted Rate; Figure S2. (a) Rural–urban (RUCC) Code Level Analysis. (b) Rural–urban (RUCC) level analysis of crude cardiovascular mortality; Table S1: Interesting counties which represent either under or over performers with respect to crude cardiovascular mortality. Table S2: Crude Mortality by RUCC Category with Number of Observations.
