# Supplementary material for: Mapping the Heartbeat of America with ChatGPT-4: Unpacking the Interplay of Social Vulnerability, Digital Literacy, and Cardiovascular Mortality in County Residency Choices
Source: J Pers Med. 2023 Nov 21;13(12):1625. doi: 10.3390/jpm13121625 (PMC10744376; doi:10.3390/jpm13121625)
Supplement: Supplementary file 1 [file jpm-13-01625-s001.zip › jpm-2673533-supplementary.pdf]

Supplemental Information

Prompt and Response S1: Example - ChatGPT 4 Code Generator Prompt

An example prompt is presented below, in this we specify our needs to the large language model (The Prompt) and in the illustration, the text below 'Chat GPT4 response' was entirely generated by Chat GPT4 including the figure. A prompt is a stimulus that triggers a specific action or event.

The Prompt

"Act like a data scientist and 1. please explore the relationship between RUCC categories, crude rate and year 2. use appropriate statistical or machine learning techniques 3. Provide illustrations and publication quality table 5. Provide description of analysis."

Chat GPT4 response

To explore the relationship between RUCC categories, crude rate, and year, we'll follow a structured approach:

Data Preparation: Load the data and prepare it for analysis.
Exploratory Data Analysis (EDA): Examine the distribution of RUCC categories, crude rates, and years in the dataset.
Statistical and Machine Learning Analysis: Apply appropriate statistical or machine learning techniques to explore the relationship.
Visualizations: Create illustrative visualizations.
Publication-Quality Table: Summarize key findings in a table format.
Description of Analysis: Summarize the methods and findings.

Let's start by loading and exploring the dataset.

1. Data Preparation

We'll begin by loading the data into a DataFrame and taking a quick look at its structure.

```
import pandas as pd

# Load the dataset
file_path = '/mnt/data/df_all_sep_14_2023.csv'
df_all = pd.read_csv(file_path)

# Show the first few rows of the dataset
df_all.head()
```

2. Exploratory Data Analysis (EDA)

Let's start by exploring the distribution of the RUCC_2013 categories, crude rates, and years in the dataset. This will help us understand the data's structure and the relationships we aim to analyze.

```
import matplotlib.pyplot as plt
```

```
import seaborn as sns

# Set up the plotting style
sns.set(style="whitegrid")

# Plot the distribution of RUCC_2013 categories
plt.figure(figsize=(10, 6))
sns.countplot(data=df_all, x='RUCC_2013', palette='husl')
plt.title('Distribution of RUCC_2013 Categories')
plt.xlabel('RUCC_2013')
plt.ylabel('Count')
plt.show()

# Plot the distribution of Crude Rate
plt.figure(figsize=(10, 6))
sns.histplot(df_all['Crude Rate'], bins=30, kde=True, color='blue')
plt.title('Distribution of Crude Rates')
plt.xlabel('Crude Rate')
plt.ylabel('Frequency')
plt.show()

# Plot the distribution of Years
plt.figure(figsize=(10, 6))
sns.countplot(data=df_all, x='Year', palette='viridis')
plt.title('Distribution of Years')
plt.xlabel('Year')
plt.ylabel('Count')
plt.xticks(rotation=45)
plt.show()
```

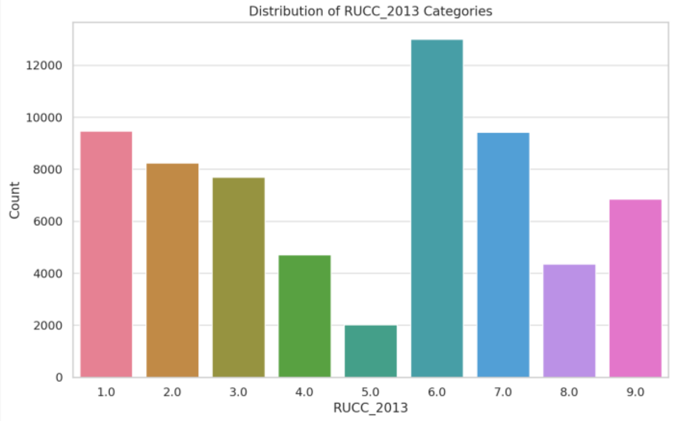

Exploratory Data Analysis (EDA) Summary:

RUCC_2013 Categories: The distribution shows varying counts for different RUCC_2013 categories. This suggests that some categories are more represented than others in the dataset.
Crude Rates: The distribution appears to be roughly bell-shaped, although we would need to perform further statistical tests to confirm its nature.
Years: The dataset spans multiple years, with varying counts for each year.

3. Statistical and Machine Learning Analysis

We'll use multiple linear regression to model how Crude Rate is influenced by Year and RUCC_2013. This technique will allow us to quantify the relationship between these variables.

4. Visualizations

We'll plot the fitted regression models to visualize how Crude Rate varies with Year for each RUCC_2013 category.
from sklearn.linear_model import LinearRegression
from sklearn.preprocessing import PolynomialFeatures

```
# Filter and forward-fill missing data
df_all['Crude Rate'] = df_all['Crude Rate'].fillna(method='ffill')

# Get unique RUCC_2013 values and sort them
unique_rucc = sorted(df_all['RUCC_2013'].dropna().unique())

# Create a color map for RUCC_2013 categories
color_map = sns.color_palette("husl", len(unique_rucc))
color_dict = {rucc: color for rucc, color in zip(unique_rucc, color_map)}

# Initialize plot
plt.figure(figsize=(12, 8))

# Initialize empty list for legend handles
legend_handles = []

# Loop over each unique RUCC_2013
for rucc in unique_rucc:
    df_rucc = df_all[df_all['RUCC_2013'] == rucc]
    summary_by_year_rucc = df_rucc.groupby('Year')['Crude
Rate'].mean().reset_index()

    # Create a mask to filter out NaN values
    mask = ~summary_by_year_rucc['Year'].isna() &
~summary_by_year_rucc['Crude Rate'].isna()

    # If all data for a RUCC_2013 is missing, continue to the next RUCC_2013
    if mask.sum() == 0:
        continue

    # Prepare data for regression model
    X = summary_by_year_rucc.loc[mask, 'Year'].values.reshape(-1, 1)
    y = summary_by_year_rucc.loc[mask, 'Crude Rate'].values.reshape(-1, 1)

    # Fit a polynomial regression model (degree 2)
    poly = PolynomialFeatures(degree=2)
    X_poly = poly.fit_transform(X)
    model = LinearRegression().fit(X_poly, y)

    # Determine the color of the plot based on RUCC_2013
    color = color_dict[rucc]

    # Plot the data points and the fitted line
    plt.scatter(X, y, color=color, alpha=0.5)
```

```
    plt.plot(X, model.predict(X_poly), color=color, linewidth=1, alpha=0.7)

    # Add legend entry
    legend_handles.append(plt.Line2D([0], [0], marker='o', color='w',
markerfacecolor=color, markersize=10, label=str(rucc)))

# Add labels and title
plt.xlabel('Year')
plt.ylabel('Crude Rate')
plt.title('Polynomial Regression: Change in Crude Rate per Year for each
RUCC_2013')

# Add sorted legend
plt.legend(handles=legend_handles, title='RUCC_2013', loc='upper right',
bbox_to_anchor=(1.3, 1.0), ncol=2)

# Show the plot
plt.show()
```

4. Visualizations Summary:

The plot visualizes how the Crude Rate varies over different Years for each RUCC_2013 category. A second-degree polynomial regression line is fitted to each group of data points to capture the trend better. The legend in the upper right corner is sorted and landscape-oriented for easier interpretation.

5. Publication-Quality Table

Let's create a summary table that encapsulates key statistics for each RUCC_2013 category, like mean and standard deviation of the Crude Rate, and the number of observations. This table will be prepared for publication quality.

6. Description of Analysis

Methods:

- Data Preparation: Missing values in the Crude Rate were forward-filled.
- Exploratory Data Analysis: Visualized the distribution of RUCC_2013, Crude Rate, and Year.
- Statistical Analysis: Applied a second-degree polynomial regression model to study the relationship between Year, Crude Rate, and RUCC_2013.

Findings:

- RUCC_2013 Distribution: Varying counts for different categories, indicating that some are more represented than others.
- Crude Rate Distribution: Appears to be roughly bell-shaped.
- Year Distribution: Varying counts for each year.
- Statistical Modeling: The polynomial regression model captures trends across RUCC_2013 categories. For example, categories 8.0 and 9.0 generally have higher crude rates and show an increasing trend over years.

These findings can be instrumental in understanding the dynamics between rural-urban settings (RUCC_2013), time (Year), and the variable of interest (Crude Rate). Further studies

can dive deeper into causal relationships and may involve more sophisticated modeling techniques.

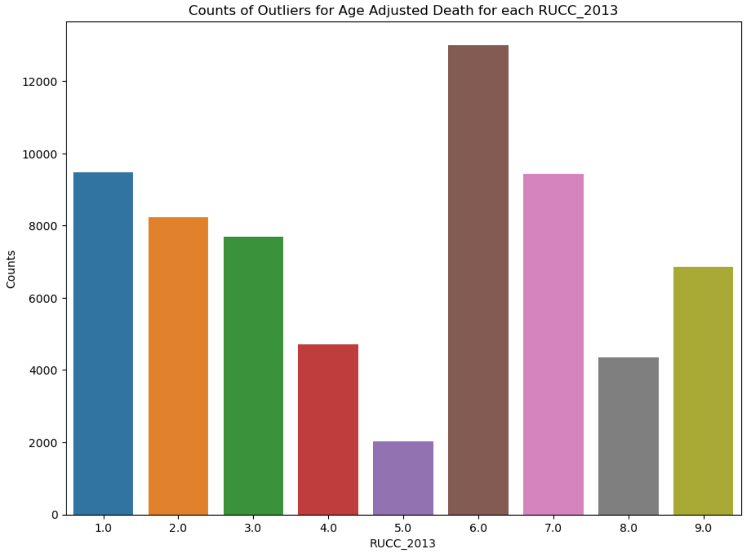

1: Metro - Counties in metro areas of 1 million population or more
2: Metro - Counties in metro areas of 250,000 to 1 million population
3: Metro - Counties in metro areas of fewer than 250,000 population
4: Nonmetro - Urban population of 20,000 or more, adjacent to a metro area
5: Nonmetro - Urban population of 20,000 or more, not adjacent to a metro area
6: Nonmetro - Urban population of 2,500 to 19,999, adjacent to a metro area
7: Nonmetro - Urban population of 2,500 to 19,999, not adjacent to a metro area
8: Nonmetro - Completely rural or less than 2,500 urban population, adjacent to a metro area
9: Nonmetro - Completely rural or less than 2,500 urban population, not adjacent to a metro area

Figure S1a: Description of Outlier Counties and Counties with Missing Values for Crude Cardiovascular mortality by RUCC status.

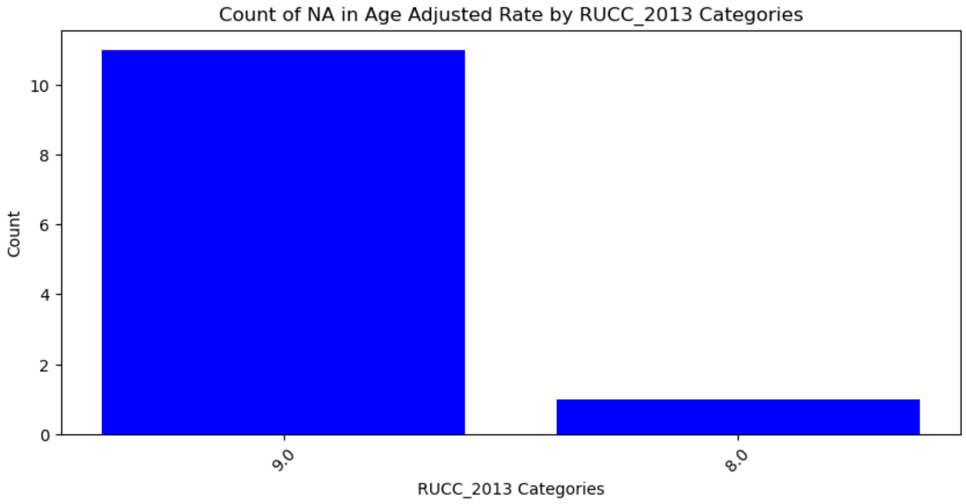

9.0: Nonmetro - Completely rural or less than 2,500 urban population, not adjacent to a metro area
8.0: Nonmetro - Completely rural or less than 2,500 urban population, adjacent to a metro area

Figure S1b: Counties with Missing Values for Age-adjusted Rate by RUCC status

Top 5 Counties Defying Expectations:

These counties have a high 'social vulnerability index' and low 'Digital_literacy',

but their 'Age Adjusted Rate' is lower than the median.

County: Dimmit County, Description: Nonmetro - Urban population of 2,500 to 19,999, adjacent to a metro area

County: Presidio County, Description: Nonmetro - Urban population of 2,500 to 19,999, not adjacent to a metro area

County: Zapata County, Description: Nonmetro - Urban population of 2,500 to 19,999, adjacent to a metro area

County: Imperial County, Description: Metro - Counties in metro areas of fewer than 250,000 population

County: DeSoto County, Description: Nonmetro - Urban population of 2,500 to 19,999, adjacent to a metro area

Table S1 Interesting counties which represent either under or over performers with respect to crude cardiovascular mortality

Top 5 Counties Performing Below Expectations:

These counties have a low 'social vulnerability index' and high 'Digital_literacy',

but their 'Age Adjusted Rate' is higher than the median.

County: King William County, Description: Metro - Counties in metro areas of 1 million population or more

County: Garfield County, Description: Nonmetro - Completely rural or less than 2,500 urban population, not adjacent to a metro area

County: Warren County, Description: Nonmetro - Completely rural or less than 2,500 urban population, adjacent to a metro area

County: Trego County, Description: Nonmetro - Completely rural or less than 2,500 urban population, not adjacent to a metro area

County: Mercer County, Description: Nonmetro - Urban population of 2,500 to 19,999, not adjacent to a metro area

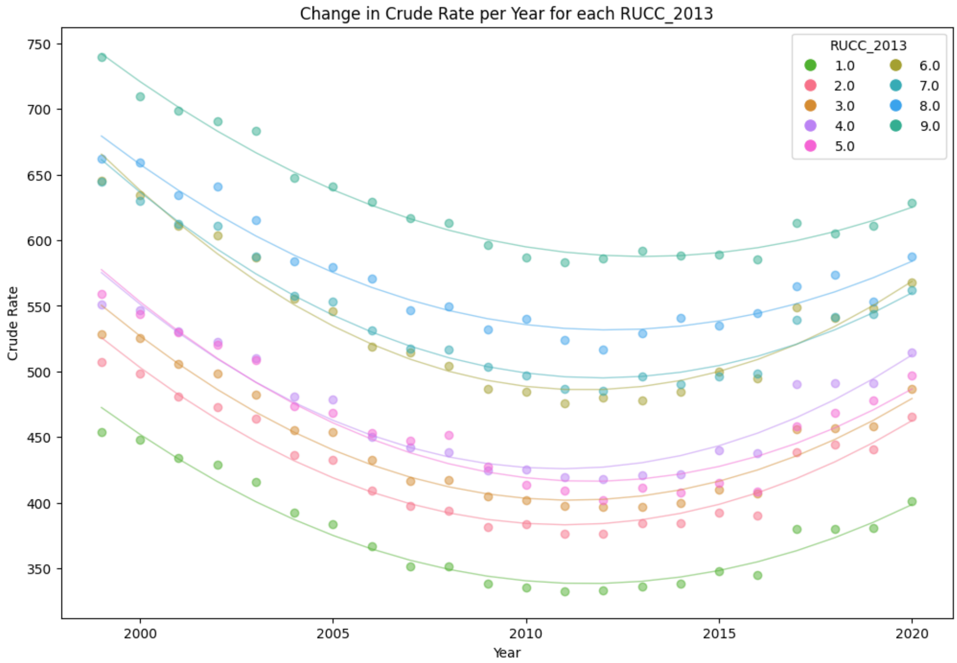

Figure S2a: Rural-Urban (RUCC) Code Level Analysis

**RUCC_2013**	**Mean Crude Rate**	**Standard Deviation**	**Number of Observations**
1.0	376.27	116.30	9475
2.0	425.23	117.49	8242
3.0	445.12	132.39	7701
4.0	470.41	122.22	4708
5.0	461.45	146.44	2019
6.0	536.88	135.58	13010
7.0	541.12	149.05	9430

8.0	572.77	138.39	4350
9.0	630.41	155.42	6856

Table S2. Crude Mortality by RUCC Category with Number of Observations

Key Metrics described in the table :

RUCC_2013: The Rural-Urban Continuum Code for the year 2013.

Mean Crude Rate: The average crude mortality rate per 100,000 population.

Standard Deviation: The standard deviation of the crude mortality rates, which provides an index of the rate variability within each RUCC category.

Number of Observations: The total number of data points collected for each RUCC category.

The data suggests a trend of increasing crude mortality rates as one moves from metropolitan to rural settings. This pattern may reflect disparities in healthcare access, social determinants of health, or other factors not captured in this dataset. The increasing standard deviation in more rural settings may indicate greater variability in mortality rates, possibly due to smaller population sizes or less consistent healthcare services.

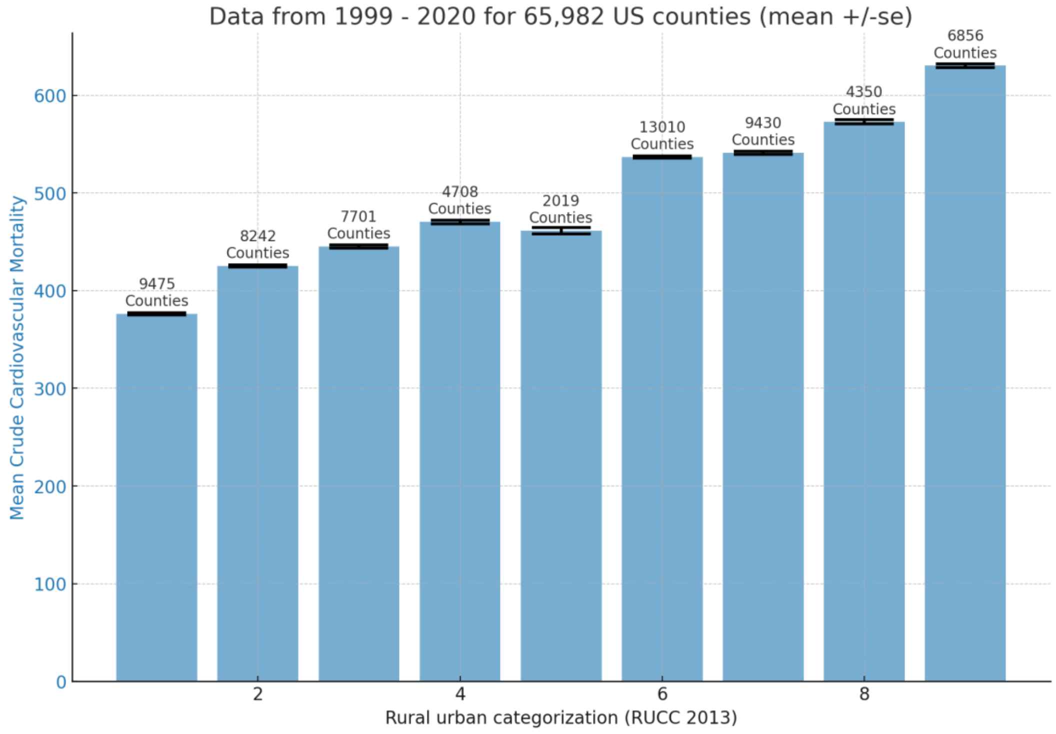

Figure S2b Rural Urban (RUCC) level analysis of crude cardiovascular mortality.
